# Resolving the intricate binding of neomycin B to multiple binding motifs of a neomycin-sensing riboswitch aptamer by native top-down mass spectrometry and NMR spectroscopy

**DOI:** 10.1093/nar/gkae224

**Published:** 2024-04-03

**Authors:** Sarah Viola Heel, Fabian Juen, Karolina Bartosik, Ronald Micura, Christoph Kreutz, Kathrin Breuker

**Affiliations:** Institute of Organic Chemistry and Center for Molecular Biosciences Innsbruck (CMBI), University of Innsbruck, Innrain 80/82, 6020 Innsbruck, Austria; Institute of Organic Chemistry and Center for Molecular Biosciences Innsbruck (CMBI), University of Innsbruck, Innrain 80/82, 6020 Innsbruck, Austria; Institute of Organic Chemistry and Center for Molecular Biosciences Innsbruck (CMBI), University of Innsbruck, Innrain 80/82, 6020 Innsbruck, Austria; Institute of Organic Chemistry and Center for Molecular Biosciences Innsbruck (CMBI), University of Innsbruck, Innrain 80/82, 6020 Innsbruck, Austria; Institute of Organic Chemistry and Center for Molecular Biosciences Innsbruck (CMBI), University of Innsbruck, Innrain 80/82, 6020 Innsbruck, Austria; Institute of Organic Chemistry and Center for Molecular Biosciences Innsbruck (CMBI), University of Innsbruck, Innrain 80/82, 6020 Innsbruck, Austria

## Abstract

Understanding small molecule binding to RNA can be complicated by an intricate interplay between binding stoichiometry, multiple binding motifs, different occupancies of different binding motifs, and changes in the structure of the RNA under study. Here, we use native top-down mass spectrometry (MS) and nuclear magnetic resonance (NMR) spectroscopy to experimentally resolve these factors and gain a better understanding of the interactions between neomycin B and the 40 nt aptamer domain of a neomycin-sensing riboswitch engineered in yeast. Data from collisionally activated dissociation of the 1:1, 1:2 and 1:3 RNA-neomycin B complexes identified a third binding motif C of the riboswitch in addition to the two motifs A and B found in our previous study, and provided occupancies of the different binding motifs for each complex stoichiometry. Binding of a fourth neomycin B molecule was unspecific according to both MS and NMR data. Intriguingly, all major changes in the aptamer structure can be induced by the binding of the first neomycin B molecule regardless of whether it binds to motif A or B as evidenced by stoichiometry-resolved MS data together with titration data from ^1^H NMR spectroscopy in the imino proton region. Specific binding of the second and third neomycin B molecules further stabilizes the riboswitch aptamer, thereby allowing for a gradual response to increasing concentrations of neomycin B, which likely leads to a fine-tuning of the cellular regulatory mechanism.

## Graphical Abstract

**Graphical Abstract:**
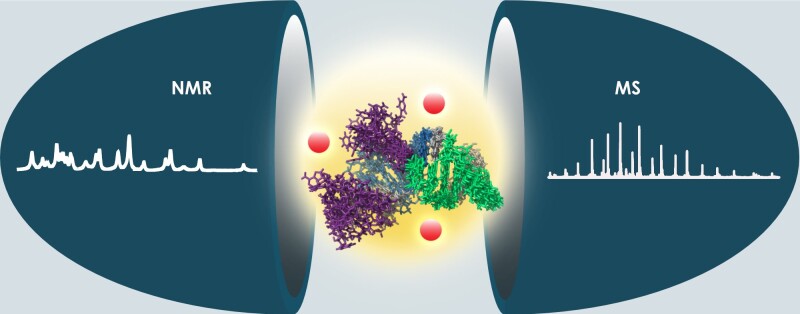

## Introduction

Ribonucleic acids (RNA) can interact with macromolecular assemblies including the ribosome (1) and RNA polymerases (2) as well as naturally occuring (3) and synthetic (4) small molecules. In recent years, the study of interactions between RNA and small molecules has gained increasing interest as researchers increasingly search for drugs that target RNA (5–12). Among these, aminoglycosides are excellent models for the study of RNA recognition (3,13) even though their positive charge at neutral pH can lead to unspecific binding to the negatively charged RNA (14,15). A number of different experimental techniques including crystallography (16–18), nuclear magnetic resonance spectroscopy (NMR) (19–22), isothermal titration calorimetry (ITC) (23,24), and native electrospray ionization (ESI) mass spectrometry (MS) (25–30) have been used for the study of RNA-aminoglycoside interactions. Multiple ligand binding (16,26,29,31–33) detected by any of these methods is often interpreted as evidence for ‘unspecific’ binding. This implies that RNAs have generally evolved a single unique binding motif for a given ligand and that 1:1 binding models can be used to describe RNA-ligand interactions. However, recent NMR (34,35) and MS (36,37) studies have challenged this view by providing evidence for the presence of multiple binding motifs for the same ligand rather than ‘unspecific’ binding to RNA. As a case in point, we have recently identified two different binding motifs of a 40 nt aptamer construct (36) of a synthetic neomycin-sensing riboswitch which showed high ligand specificity for neomycin B and dose-dependent regulation of gene expression in yeast (38,39). However, identifying multiple binding motifs of an RNA and determining their relative occupancies with small molecule ligands is not trivial, and so far we have only reported the analysis of native top-down MS data for 1:1 complexes (36). Here, we extend our native top-down MS approach to complexes of the riboswitch aptamer with up to four neomycin B molecules and complement the MS data with data from ^1^H NMR spectroscopy in the imino proton region.

**Scheme 1.**
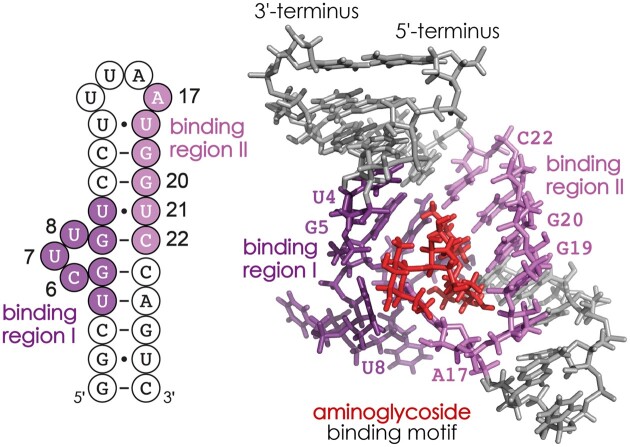
Neomycin B binding to RNA **1** determined by CAD MS (36) illustrates how two different binding regions (I: U4–U10 in dark violet, II: A17–C22 in light violet) that are distant in sequence (left) form a binding motif for the aminoglycoside ligand (red) in the NMR structure 2MXS (19) (right).

More specifically, we demonstrate how data from native ESI MS in combination with stoichiometry-resolved, low-energy collisionally activated dissociation (CAD) (40) can be used to localize different binding regions of RNA complexes with two (1:2) and three (1:3) ligand molecules. Binding regions are stretches of nucleotides that are adjacent to each other in sequence and to which a ligand molecule is bound. With the aid of structure prediction for the free RNA using the MC-fold|MC-Sym pipeline (41), the different binding regions can then be interpreted in terms of binding motifs. Binding motifs are a combination of typically two different binding regions that are distant from each other in sequence but sufficiently close in space that a neomycin B molecule can bind to both regions (Scheme 1). Moreover, we show how the MS data can be used to derive relative occupancies with neomycin B of the different binding motifs for each stoichiometry. Next, we use ^1^H NMR spectroscopy in the imino proton region to confirm the structure prediction for the free RNA and to follow the changes in RNA structure induced by neomycin B binding in titration experiments. Finally, the evidence from MS and NMR experiments is combined to obtain a clearer picture of how many neomycin B molecules bind to the riboswitch aptamer, where they bind and with what occupancy, and how this affects the riboswitch aptamer structure.

## Materials and methods

RNAs **2**–**5** (Table 1) for mass spectrometry experiments were prepared by solid phase synthesis, purified by HPLC, and desalted using centrifugal concentrators (Vivaspin 500, MWCO 3000, Sartorius AG, Germany) (42). ^15^N-labeled RNA **2** for NMR experiments was prepared in the same way but used in-house synthesized [1,3-^15^N_2_]uridine and [1-^15^N]guanosine phosphoramidites (43) for incorporation at positions 4, 7, 23 and 32 ([1-^15^N]G4, [1,3-^15^N_2_]U7, [1-^15^N]G23 and [1,3-^15^N_2_]U32). RNA concentration was determined by UV absorption at 260 nm using an Implen Nano Photometer (Implen, Germany). Prior to MS or NMR experiments, RNA solutions were heated to 90°C for 2 min followed by rapid cooling on ice. Sulfate salt of neomycin B, piperidine, piperazine, spermidine, ammonium acetate, and ammonium bicarbonate were purchased from Sigma-Aldrich (Vienna, Austria). H_2_O was purified to 18 MΩ·cm at room temperature using a Milli-Q system (Millipore, Austria) and CH_3_OH (VWR, Austria) was HPLC-grade.

**Table 1.** RNA studied (all OH-terminated); conserved sequence underlined and bold

RNA	sequence	nt
**1**	5′- GGCU**GCUUGUCCUUUAAUGGUCC**AGUC -3′	27
**2**	5′-CCGGCAUA**GCUUGUCCUUUAAUGGUCC**UAUGUCGAAAAUG-3′	40
**3**	5′-CCGUCAUA**GCUUGUCCUUUAAUGGUCC**UAUGACGAAAAUG-3′	40
**4**	5′-CCCUGAUA**GCUUGUCCUUUAAUGGUCC**UAUCUGGAAAAUG-3′	40
**5**	5′-CCGGCAUA**GCUU**C**UCCUUUAAUGGUCC**UAUGUCGAAAAUG-3′	40

MS experiments with RNAs **2**–**4** were performed on a 7 T Fourier transform-ion cyclotron resonance (FT-ICR) instrument (Apex Ultra, Bruker, Austria) equipped with an ESI source, a linear quadrupole for ion isolation, a collision cell for CAD, and an ICR cell for ion detection. Solutions with desalted RNA (1 μM) and neomycin B (1–5 μM) in 9:1 H_2_O/CH_3_OH with 50 mM ammonium bicarbonate and 0.25 mM piperazine (pH ∼7.5) were incubated for 3 hours prior to ESI at a flow rate of 1.5 μl/min. We chose ammonium bicarbonate for ESI because it is volatile, buffers at physiological pH, and because the bicarbonate buffer system is found in blood, tear fluid, and the kidneys among other tissues. The buffer concentration of 50 mM is very close to that used in our NMR experiments and mid-range of the 10–100 mM concentration range typically used for NMR of RNA (44). As in the experiments for the determination of the NMR structures of RNA **1** in complexes with paroromomycin and ribostamycin (pdb entries 2MXS and 2N0J) by Wöhnert *et al.* (19), no divalent ions were added to the solutions used for ESI; the addition of 10 mM MnCl_2_ to a solution of RNA **1** with ribostamycin caused only line broadening of the NMR signals but no changes in chemical shift (22). ESI spectra were obtained by operation of the linear quadrupole in transmission (radiofrequency-only) mode. For CAD, ions of interest were isolated in the quadrupole and dissociated in the collision cell using laboratory frame collision energies of 137–150, 143 and 150–156 eV for CAD of (RNA + 1·neomycin B – 13H)^13−^, (RNA + 2·neomycin B – 13H)^13−^ and (RNA + 3·neomycin B – 12H)^12−^ ions, respectively. For ESI and CAD, 25–100 and 500 scans were added for each spectrum, respectively. MS experiments with RNA **5** were performed on a Fusion Lumos Orbitrap instrument (Thermo Scientific, Austria) kindly made available to us by the Institute of Biochemistry of the University of Innsbruck. The energies used for CAD in the ion routing multipole of the Orbitrap instrument were adjusted such that the yields of ***c*** and ***y*** fragments from dissociation of 1:1, 1:2, and 1:3 complexes of RNA **2** with neomycin B were similar (±2%) to those obtained with the FT-ICR instrument. Data reduction utilized the SNAP2 algorithm (Bruker, Austria) or FAST MS, a software programmed in our group by Michael Palasser (45), as well as manual inspection of the spectra. Relative ion abundances were calculated from the corresponding signal heigths in the spectra, divided by the number of charges as ICR detector response is proportional to this number (46). Only signals with a signal-to-noise ratio >3 and a mass accuracy <5 ppm were considered for analysis.

For NMR experiments, ^15^N-labeled RNA **2** was lyophilized as a sodium salt, dissolved in 450 μl NMR buffer (15 mM sodium phosphate, 25 mM NaCl, 0.1% NaN_3_, pH 6.5, 10% D_2_O) to a concentration of 280 μM, and transferred into standard 5 mm NMR tubes. NMR spectra were acquired at 15°C on a Bruker 700 MHz Avance Neo NMR equipped with a Prodigy TCI probe. Imino proton signal assignment was achieved by 2D ^1^H-^1^H jump and return NOESY correlation experiments. Starting points (G4, U7, G23 and U32) for the assignment procedure were determined from the site-specifically ^15^N-labelled RNA by running a ^1^H–^15^N-SOFAST HSQC pulse sequence. All datasets were processed with Topspin 4.2.0 (Bruker Biospin) and imported to POKY for the resonance assignment.

For structure prediction of the free RNA, we used the MC-fold|MC-Sym pipeline (41) because it is open access (https://www.major.iric.ca/MC-Pipeline/), predicts RNA secondary and tertiary structures including non-canonical base pairs, and because its predictive power was evaluated against experimental structures. However, other programs (47,48) are available that can also serve the purpose of providing a general idea about the structure of the free RNA.

## Results and discussion

**Scheme 2.**
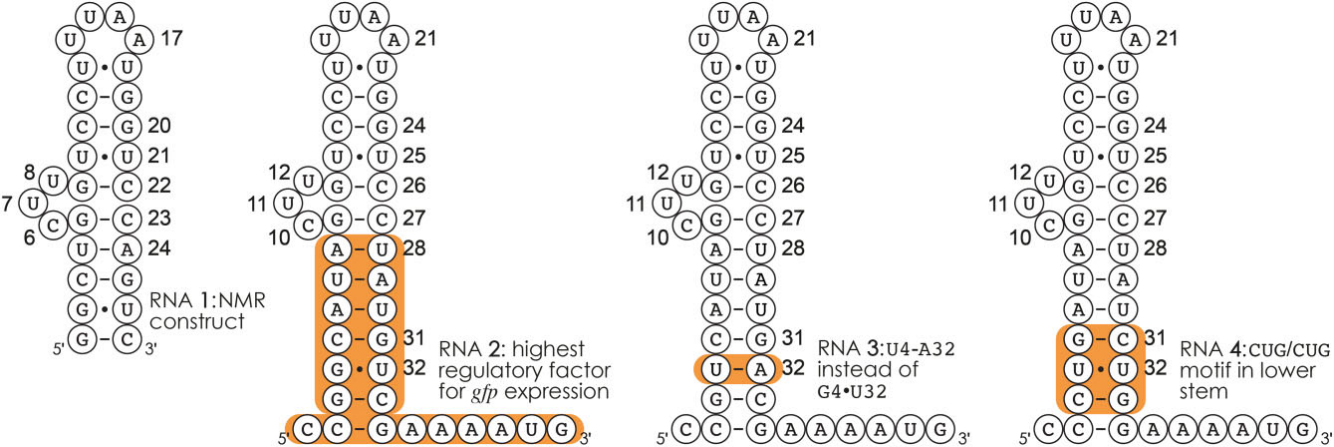
RNAs **2**–**4** studied here are the aptamers RNA **2** that showed the highest regulation for *gfp* expression in riboswitch activity studies, and two constructs in which G4•U32 of RNA **2** was replaced by U4-A32 (RNA **3**) and G3/G4/C5 and G31/U32/C33 were replaced by C3/U4/G5 and C31/U32/G33 (RNA **4**); the NMR construct RNA **1** is shown for comparison. RNAs **1**, **3**, and **4** have not yet been tested for riboswitch activity.

In our previous study (36), we demonstrated how CAD MS can be used to identify RNA binding motifs and to quantify their relative occupancy with ligand for 1:1 RNA-ligand complexes. Here we show how to extend the analysis of data from CAD MS to complexes with higher stoichiometry (1:2 and 1:3), and complement the MS data with data from NMR spectroscopy. The RNAs studied first (Scheme 2) were RNA **2**, which is the neomycin-sensing riboswitch aptamer that showed the highest regulatory factor for *gfp* expression among related sequences in riboswitch activity studies (38,39), and two RNAs designed for our MS studies in which G4•U32 of RNA **2** was replaced by U4-A32 (RNA **3**) and G3/G4/C5 and G31/U32/C33 were replaced by C3/U4/G5 and C31/U32/G33 (RNA **4**) (36). Experimental structures of RNAs **2**–**4** are not available, but structures of the NMR construct RNA **1** in complexes with paromomycin and ribostamycin (pdb entries 2MXS and 2N0J, respectively) have been published (19).

**Scheme 3.**
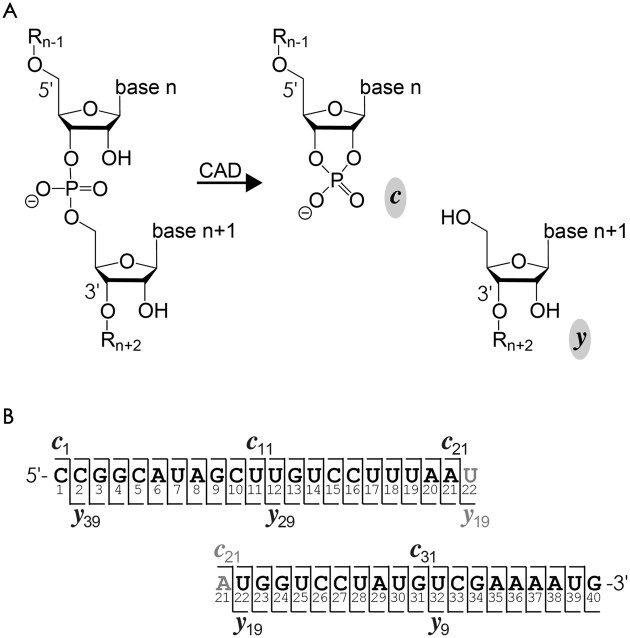
(**A**) RNA phosphodiester backbone bond cleavage by CAD produces ***c*** and ***y*** fragments; (**B**) fragment ion numbering illustrated in a cleavage map for RNA **2**.

**Figure 1.**
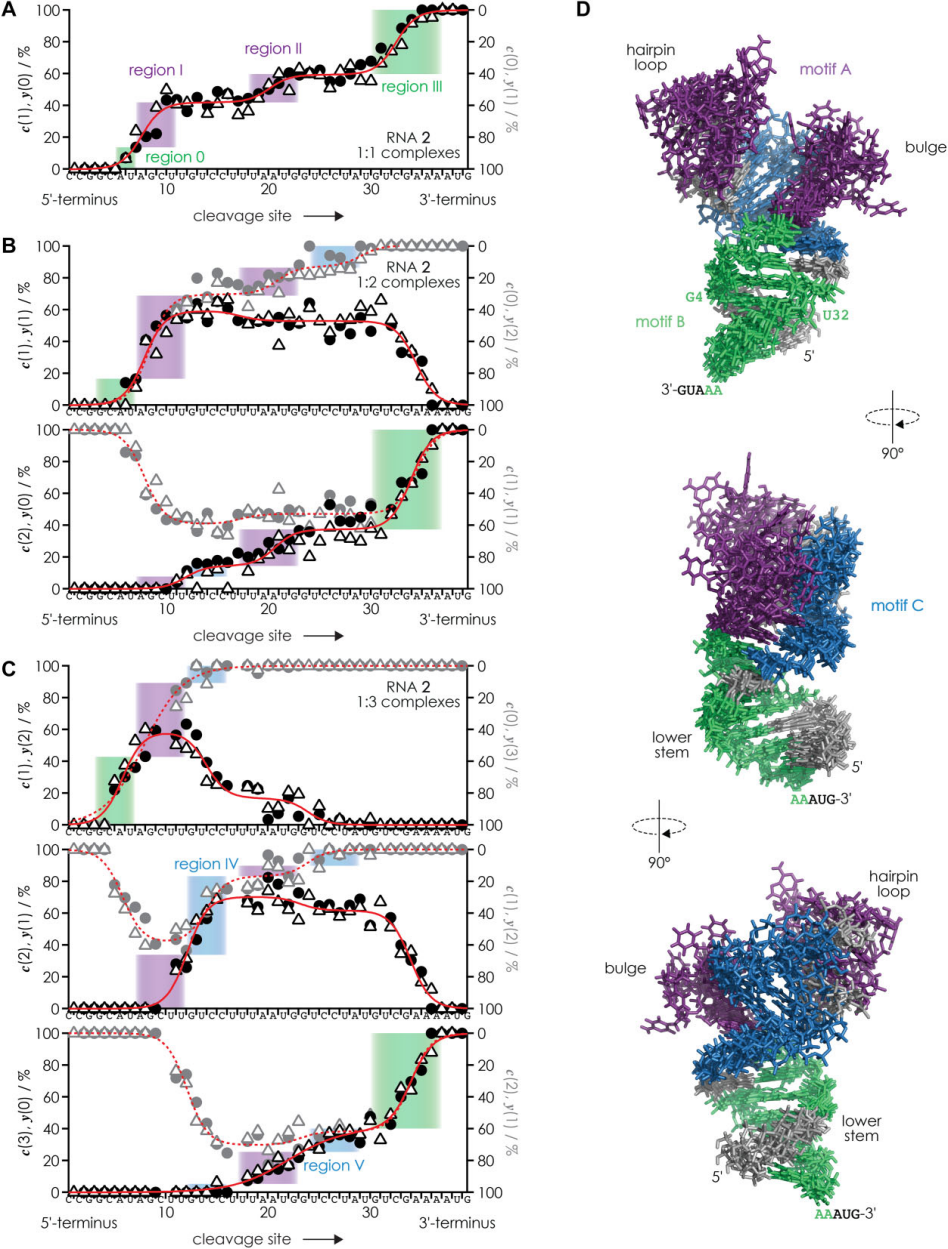
Percentage of *c* (circles) and *y* (triangles) fragments with 0, 1, 2 or 3 neomycin B molecules attached versus RNA cleavage site from CAD of (**A**) (RNA **2** + 1·neomycin B – 13H)^13−^ ions, (**B**) (RNA **2** + 2·neomycin B - 13H)^13−^ ions and (**C**) (RNA **2** + 3·neomycin B – 12H)^12−^ ions with single, double, or triple sigmoidal fit functions shown as red lines; (**D**) binding regions of the 1:2 and 1:3 complexes mapped onto five structures predicted for free RNA **2** (excluding A36–G40) by the MC-fold|MC-Sym pipeline (41) indicate binding motifs A (formed by the hairpin loop and the bulge), B (in the minor groove of the lower stem, stabilized by interactions with the first nucleotides of the overhang) and C (in the minor groove of the upper stem) highlighted in violet, green and blue, respectively. The data in A) and B) were previously published in (36) but without the analysis shown here.

Collisionally activated dissociation of 1:1, 1:2 and 1:3 complexes of RNAs **2**–**4** with neomycin B produced ***c*** and ***y*** fragments from phosphodiester backbone bond cleavage (40,49,50) (Scheme 3) without and with up to 3 neomycin B molecules attached, which we refer to in the following as ***c***(0), ***c***(1), ***c***(2), ***c***(3), and ***y***(0), ***y***(1), ***y***(2), ***y***(3). Site-specific percentages of ***c***(m) fragments without and with up to three neomycin B molecules attached (*m* = 0–3) were calculated relative to all ***c*** fragments from a given cleavage site; percentages of ***y***(m) were calculated accordingly. Loss of neomycin B was a minor dissociation channel (<2%), so that for each cleavage site, the percentages of fragments that are complementary with respect to the number of neomycin B molecules attached are the same within experimental error (Figure 1A–C). In other words, all neomycin B molecules are accounted for and can be found on either the ***c*** or the ***y*** fragment from RNA backbone cleavage at a given site. For the 1:1 complexes (m = 1), these are ***c***(0) and ***y***(1) as well as ***c***(1) and ***y***(0), for the 1:2 complexes (m = 2) ***c***(0) and ***y***(2), ***c***(1) and ***y***(1) as well as ***c***(2) and ***y***(0), and for the 1:3 complexes (*m* = 3) ***c***(0) and ***y***(3), ***c***(1) and ***y***(2), ***c***(2) and ***y***(1) as well as ***c***(3) and ***y***(0). In Figure 1A–C and Supplementary Figure S1 and S2, the data for complementary ***c*** and ***y*** fragments are shown as separate symbols for clarity, but we used both for nonlinear least squares fitting with single, double, or triple sigmoidal functions, the residuals of which indicate standard deviations of ∼5% (Supplementary Figures S1 and S2).

With the normalization described above, occupancy values of 100% indicate a fully occupied binding motif, and occupancy values <100% indicate a partially occupied binding motif. However, as binding motifs are composed of binding regions (Scheme 1), occupancy values >100% can result for the 1:2 and 1:3 complexes as shown further below. Occupancy values >100% indicate that two neomycin B molecules bind to a motif in a fraction of the complexes, which can be the case if the binding motif is large and/or flexible enough to accommodate two neomycin B molecules.

To explain in detail how we developed top-down MS for the identification of RNA binding motifs and their relative occupancy with ligand in 1:2 and 1:3 complexes, we first summarize the analysis and interpretation of data from CAD of 1:1 complexes of RNA **2** with neomycin B published recently (36). Figure 1A shows the percentages of ***c***(1) fragments with and complementary ***y***(0) fragments without neomycin B attached versus RNA cleavage site. Because the percentages of ***c***(0) and ***c***(1)—and those of ***y***(0) and ***y***(1)—add up to 100% for the 1:1 complexes, plotting the percentages of ***c***(0) and ***y***(1) on a reversed axis (right) gives the same picture. In the following, we discuss the data from the 5′ to the 3′ terminus. The percentages of ***c***(1) and ***y***(0) fragments were zero for cleavage sites 1–5, i.e. for ***c***_1_–***c***_5_ and ***y***_35_–***y***_39_ (Scheme 3), indicating that neomycin B does not bind to C1–C5. The percentages of ***c***(1) and ***y***(0) sigmoidally increased from site 5 to site 11, indicating binding of neomycin B to A6–U11. After a plateau at sites 11–18, indicating no binding of neomycin B to U12–U18, the percentages of ***c***(1) and ***y***(0) further increased to ∼60% at site 23, indicating binding of neomycin B to U19–G23. Another plateau at sites 23–30 and another increase at sites 30–37 suggest no binding of neomycin B to G24–U30 and binding of neomycin B to G31–A37, respectively. Finally, the 100% values for ***c***(1) and ***y***(0) at sites 37–39 show that neomycin B does not bind to A38–G40. From a comparison with data from CAD of 1:1 complexes of RNA **1**, we postulated that the sequence stretch A6–U11 comprises two adjacent binding regions, A6–U7 (region 0) and A8–U11 (region I) (36). Further, U19–G23 and G31–A37 were assigned as binding regions II and III, respectively. Mapping the binding regions 0-III onto the calculated structure of free RNA **2** (Figure 1D) reveals two separate binding motifs A (regions I and II) and B (regions 0 and III). Note that both binding motifs consist of binding regions that are distant in sequence, meaning that for the separation of fragments from RNA backbone cleavage in and between the regions of a given motif, CAD must also have dissociated some of the noncovalent bonds between the aminoglycoside and the RNA, leaving it bound to either the ***c*** or the complementary ***y*** fragment (36). Importantly, the heights of the transitions for each binding region (highlighted by colored rectangles in Figure 1A–C) reflect the relative occupancy of the different binding motifs. For the 1:1 complexes, the increase in the percentages of ***c***(1) and ***y***(0) for binding regions I and II (the hairpin loop-bulge motif A, violet rectangles) add up to 46% ± 9%, and those for binding regions 0 and III (minor groove motif B in the lower stem, green rectangles) add up to 54% ± 9%. Thus in the 1:1 complexes of RNA **2** with neomycin B, binding motifs A and B are approximately equally occupied.

Figure 1B shows data from CAD of 1:2 complexes of RNA **2** with neomycin B (*m* = 2). The upper graph shows site-specific percentages of ***c***(1) and complementary ***y***(1) plotted as black symbols on the left axis, and the percentages for ***c***(0) and complementary ***y***(2) plotted as gray symbols on the reversed right axis. The lower graph in Figure 1B shows percentages of ***c***(2) and complementary ***y***(0) plotted as black symbols on the left axis, and the percentages for ***c***(1) and complementary ***y***(1) plotted as gray symbols on the reversed right axis. The major sigmoidal transitions in Figure 1B are in the same positions as in Figure 1A, which shows that the majority of neomycin B molecules occupy binding motifs A and B in the 1:2 complexes. However, transitions at sites 12–16 and 24–29 (blue rectangles) show that some of the neomycin B molecules bind to regions other than those in the 1:1 complexes, which we assign to yet another binding motif C in the minor groove of the upper stem of RNA **2** (Figure 1D).

For determining the relative occupancies of the different binding motifs in the 1:2 complexes, we follow the traces in Figure 1B that range from 0% to 100% (each corresponding to one neomycin B molecule), i.e. the trace for ***c***(0) and ***y***(2) in the upper plot and the trace for ***c***(2) and ***y***(0) in the lower plot of Figure 1B. Adding the percentage values for motif A (regions I and II, violet rectangles), motif B (regions 0 and III, green rectangles), and motif C (regions IV and V, blue rectangles) in Figure 1B shows that in the 1:2 complexes, motif A is fully occupied (100%) and that motifs B and C are occupied to ∼80% and ∼20%, respectively.

The three plots illustrating the CAD data for neomycin B binding to RNA **2** in the 1:3 complexes (Figure 1C) show that the positions of the transitions and thus the binding regions were the same as for the 1:2 complexes (Figure 1B). For determining the relative occupancies of the different binding motifs, we again follow the traces that range from 0% to 100%, i.e.those for ***c***(0) and ***y***(3) in the upper plot, and ***c***(3) and ***y***(0) in the lower plot of Figure 1C. In the middle plot, neither trace ranges from 0% to 100%, but because they overlap in region IV (G13-C16), we can combine the trace for ***c***(2) and***y***(1) at sites 1–14 (0–60%, solid red line) with the trace for ***c***(1) and ***y***(2) at sites 14–39 (60–100%, dashed red line). Adding the percentage values for motif A (regions I and II, violet rectangles), motif B (regions 0 and III, green rectangles), and motif C (regions IV and V, blue rectangles) in Figure 1C shows that in the 1:3 complexes, each motif is, within error limits, fully occupied.

**Figure 2.**
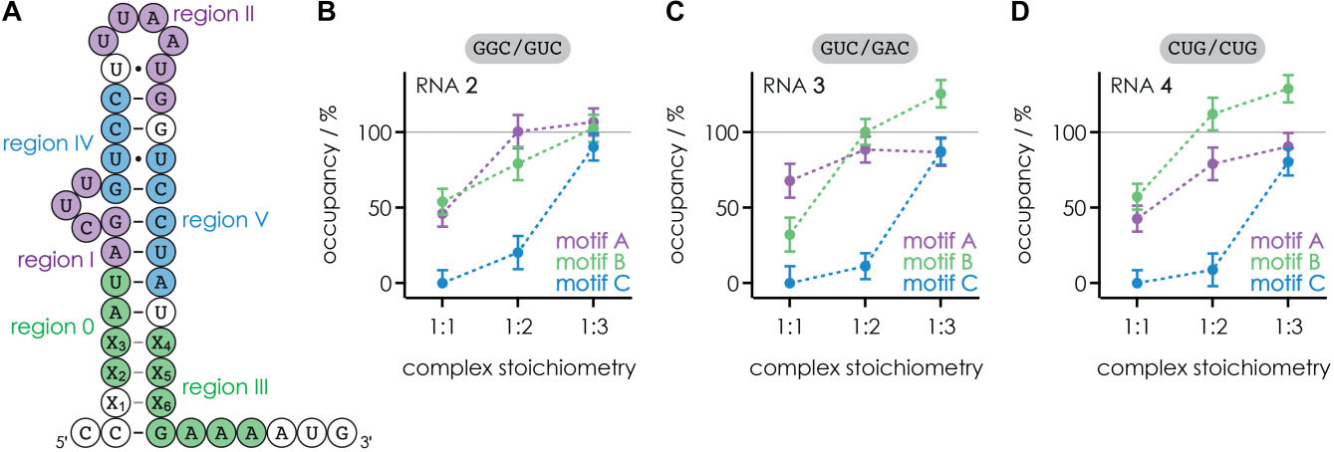
A) Binding motifs A (regions I and II, violet), B (regions 0 and III, green) and C (regions IV and V, blue) of the 1:3 complexes mapped onto the predicted secondary structure of RNA **2** and their occupancies derived from the CAD data in Figures 1, Supplementary Figures S3, and S4 for 1:1, 1:2 and 1:3 complexes of B) RNA **2** (X_1_X_2_X_3_/X_4_X_5_X_6_ = GGC/GUC), C) RNA **3** (GUC/GAC), D) RNA **4** (CUG/CUG); errors were calculated from standard deviations of the residuals of fit functions of the corresponding CAD data including error propagation (Supplementary Figure S1 and S2). Each neomycin B molecule in the stoichiometry of a complex corresponds to 100% occupancy, i.e. the occupancies of motifs A-C in the 1:1, 1:2, and 1:3 complexes add up to 100%, 200% and 300%, respectively. Overoccupancy (values > 100%) of motif B indicates binding of two neomycin B molecules to the lower stem in a fraction of the complexes (∼25% in the 1:3 complexes of RNA **3**; ∼10% and ∼30% in the 1:2 and 1:3 complexes of RNA **4**, respectively).

Figures 2B summarizes the occupancies of binding motifs A, B and C of RNA **2** for all complex stoichiometries studied. We also studied the 1:1, 1:2, and 1:3 complexes of RNAs **3** and **4** with neomycin B (Supplementary Figures S3 and S4), for which the corresponding occupancies are shown in Figures 2C and D, respectively. In the 1:1 complexes of RNA **2**, binding motifs A and B are approximately equally occupied, and neomycin B does not bind to motif C. The addition of a second neomycin B molecule saturates binding motif A in the 1:2 complexes of RNA **2**, increases binding to motif B from ∼55% to ∼80%, and results in ∼20% occupancy of motif C. In the 1:3 complexes of RNA **2**, all three motifs are, within error limits, fully occupied.

RNA **3** contains the canonical base pair U4–A32, with A32 being part of binding motif B, instead of the noncanonical base pair G4•U32 of RNA **2** (Scheme 2). In the 1:1 complexes of RNA **3**, neomycin B again does not bind to motif C, and binding motifs A and B are ∼70% and ∼30% occupied, respectively (Figure 2C). This observation is consistent with reduced binding of neomycin B to the lower stem of RNA **3** in the absence of the noncanonical base pair G4•U32. However, despite the reduced affinity of motif B of RNA **3** for neomycin B, binding motif A is not fully occupied in the 1:2 and 1:3 complexes, which suggests that G4•U32 of RNA **2** in some way stabilizes neomycin B binding to motif A. While neither motif A nor C are fully occupied in the 1:3 complexes of RNA **3**, binding motif B is overoccupied at ∼125%, indicating that in ∼25% of the 1:3 complexes, two neomycin B molecules are bound to the lower stem (motif B), but their exact locations cannot be resolved from the data in Supplementary Figure S3.

In RNA **4**, we replaced three adjacent base pairs in the lower stem of RNA **2** with CUG/CUG (51), reintroducing a noncanonical base pair at the original position (albeit a different one, U4•U32 instead of G4•U32, Figure 2D) framed by stable G–C base pairs (Scheme 2). This mutation extended binding region 0 to U4 in the 1:1 complexes, and increased neomycin B binding to motif B for all complex stoichiometries when compared to RNA **2**. Accordingly, neither motif A nor C are fully occupied in any of the complexes of RNA **4**, and in ∼30% of the 1:3 complexes, two neomycin B molecules are bound to the lower stem (motif B). The overoccupancy of motif B for RNAs **3** (∼125%) and **4** (∼130%) is in striking contrast to RNA **2**, for which each binding motif was, within error limits, occupied to 100%. This indicates a heterogeneous binding pattern for RNAs **3** and **4**, with two neomycin B molecules bound to the lower stem in a fraction (∼25% and ∼30%) of the 1:3 complexes, whereas a homogeneous binding pattern is found for the 1:3 complexes of RNA **2**.

The major findings from the above data from CAD of 1:1, 1:2, and 1:3 complexes of RNAs **2**–**4** with neomycin B are that (i) binding motif A is fully occupied only in the 1:2 and 1:3 complexes of RNA **2** which has the sequence with the highest regulatory factor for riboswitch function (38), (ii) binding motif B is overoccupied in the 1:2 complexes of RNA **3** and the 1:3 complexes of RNAs **3** and **4**, (iii) replacing the noncanonical base pair G4•U32 with the canonical base pair U4–A32 in RNA **3** decreases the occupancy of binding motif B in the 1:1 but not the 1:2 and 1:3 complexes and (iv) the occupancy of binding motif C is not significantly affected by the sequence mutations in the lower stem. Because the central binding motif A—whose residues are part of the consensus sequence of the functional riboswitch—is fully occupied only in RNA **2**, and motif B is overoccupied in RNAs **3** and **4**, we conclude that RNA **2** has the highest specificity for neomycin B binding, even though the overall binding affinity for neomycin B is the same for RNAs **2**–**4** within error limits (36).

**Figure 3.**
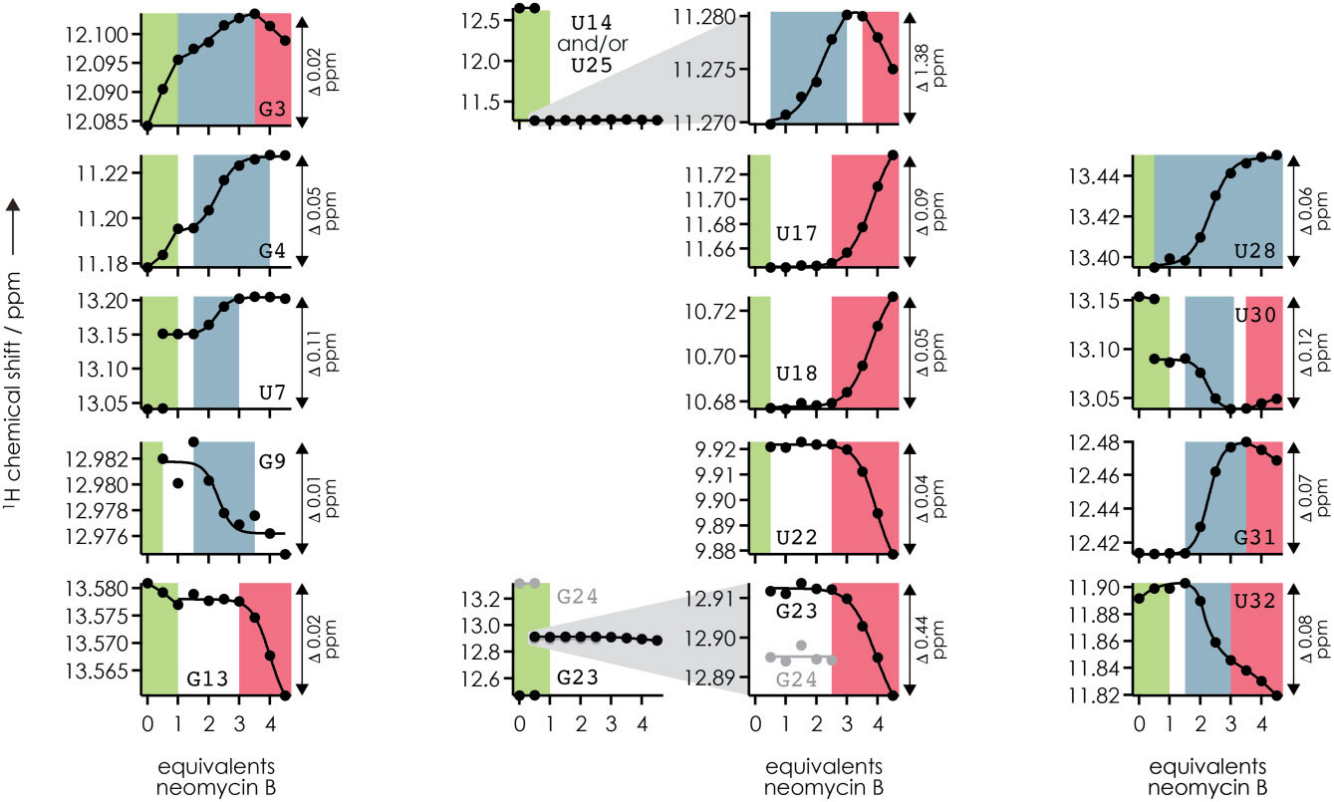
^1^H chemical shifts in the imino proton region for individual residues of RNA **2** (from Supplementary Figure S5) plotted versus the equivalents of neomycin B in the solutions used for NMR spectroscopy; solid lines are meant to guide the eye, and the ranges in which distinct changes in ^1^H chemical shifts were observed are color coded in limegreen, gray-blue, and red.

**Figure 4.**
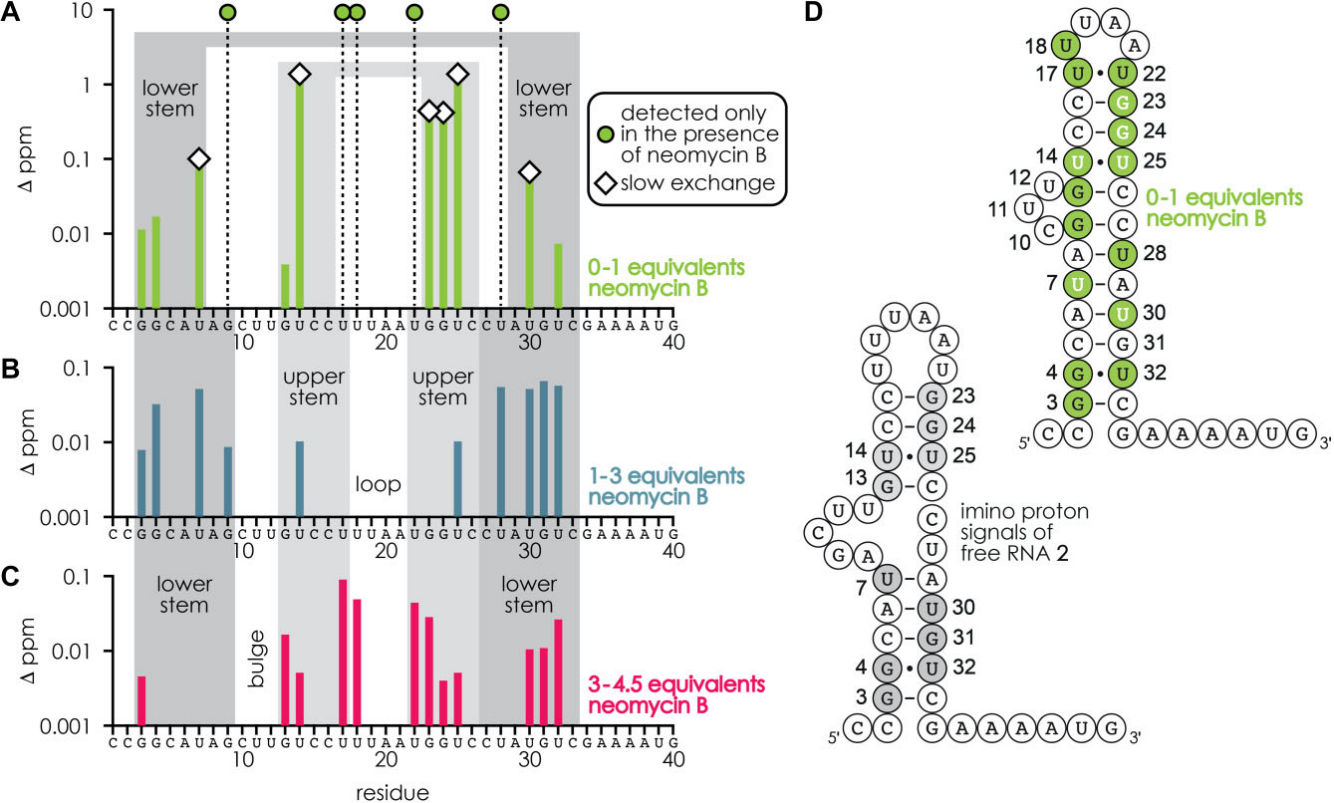
Residue-specific changes in imino proton ^1^H chemical shifts of RNA **2** (from Supplementary Figure S5) plotted on a logarithmic scale for (**A**) 0 to 1, (**B**) 1 to ∼3 and (**C**) ∼3 to 4.5 equivalents of neomycin B as indicated in Figure 3 and (**D**) proposed secondary structures of free (bottom left; residues which showed imino proton signals highlighted in gray) and bound (top right; residues which showed changes in imino proton signals in the 0–1 equivalent range are highlighted in limegreen and residues which showed slow exchange are highlighted with white letters) RNA **2**.

To experimentally test the structure of free RNA **2** predicted by the MC-fold|MC-Sym pipeline (41) (Figure 1D) and to see how neomycin B binding affects the structure of RNA **2**, we used ^1^H NMR spectroscopy in the imino proton region. Spectra were recorded from solutions of RNA **2** with 0–4.5 equivalents neomycin B (Supplementary Figure S5), and the data are summarized in Figures 3 and 4. Figure 3 shows the chemical shifts of individual residues versus added neomycin B equivalents, which immediately reveal distinct changes in the lower (0–1 equivalents), intermediate (approximately 1–3 equivalents), and higher (approximately 3–4.5 equivalents) equivalent ranges studied, highlighted in limegreen, gray-blue, and red, respectively. The by far largest changes in the imino proton spectra were observed in the 0–1 equivalent range (Figure 4). In the absence of neomycin B, the imino proton signals indicate base pairing of G3, G4, U7, U30, G31, U32 in the lower stem and G13, U14, G23, G24, U25 in the upper stem of RNA **2** (Supplementary Figure S5), in good agreement with the structure of free RNA **2** predicted by the MC-fold|MC-Sym pipeline (41) (Figures 1D and 4D). The observation of imino proton resonances for G13-C26 and U14•U25 suggests that the free RNA **2** is better prestructured for neomycin B binding than the NMR construct RNA **1**, for which the corresponding base pairs showed no (G9-C22) or only very broad (U10•U21) imino proton resonances (39). Five new signals (G9, U17, U18, U22, U28) appeared with the addition of 0.5 equivalents of neomycin B to RNA **2** (Supplementary Figure S5, 3), suggesting the formation of nucleobase interactions of U18 in the hairpin loop and extension of both the upper (U17-U22) and lower (A8-U28, G9-C27) stems; stabilization of the hairpin loop and the upper stem by aminoglycoside binding has also been observed for RNA **1** (which lacks the lower stem of RNA **2**) in previous NMR (19) and chemical probing MS (45) studies. Moreover, five imino proton signals (U7, U30, G23, G24, U14/U25; the latter two residues give overlapping resonances) showed evidence for slow exchange between the free and bound forms of RNA **2** on the chemical shift time scale (Supplementary Figure S5, 3, 4) indicating high affinity binding. Importantly, both the signals that were only detected in the presence of neomycin B and the signals that showed evidence for high affinity binding were from residues in both the upper and lower parts of RNA **2** (Figure 4D), which is difficult to explain by neomycin B binding to a single binding motif for geometric reasons. The NMR data, which furthermore show chemical shift changes for G3, G4, G13 and U32 in the 0–1 equivalent range (Figure 4A), are instead consistent with neomycin B binding to two different binding motifs as indicated by CAD MS (Figure 1A). In other words, the NMR data strongly suggest that the 1:1 complexes of RNA **2** in bulk solution are a mixture of two distinct complexes with neomycin B bound to either motif A or motif B. Although the fractions of 1:1 complexes with neomycin B bound to motifs A and B cannot be deduced from the NMR data in Supplementary Figure S5, the data from CAD MS (Figures 1A and 2A) show that motifs A and B are equally occupied.

At elevated neomycin B concentrations, only smaller (<0.1 ppm) changes in chemical shift were observed (Figure 4B, C), suggesting that all major changes in the structure of RNA **2** were already induced by the binding of the first neomycin B molecule, regardless of whether it binds to motif A or B. Now the question arises, how can binding of neomycin B to motif A have a similar effect on the overall structure of RNA **2** as binding of neomycin B to motif B? In other words, how can neomycin B binding to motif A affect the structure of RNA **2** in the lower stem, and how can neomycin B binding to motif B affect the structure of RNA **2** in the upper stem? A possible rationale for this is that the formation of base pairs in the lower stem (A8–U28, G9–C27, Figure 4A) induced by neomycin B binding to motif B stabilizes U14•U25 (whose imino protons showed by far the largest change in chemical shift among all residues, Figure 3) which in turn could stabilize the upper stem of RNA **2**. Likewise, binding of neomycin B to motif A induces the formation of U17–U22 (Figure 4A) and stabilizes U14•U25 in the upper stem which in turn could stabilize the lower stem of RNA **2**. In this way, U14•U25 could act as an allosteric relay for stabilization of the entire riboswitch aptamer structure regardless of whether the first neomycin B molecule binds to motif A or B. The chemical shift changes in the range of approximately 1–3 equivalents can then be interpreted as binding of a second neomycin B molecule such that motifs A and B are populated to the extent indicated in Figure 2A (motif A: ∼100%, motif B: ∼80%) but without causing major structural changes, in agreement with a two-step binding process as proposed by Sigurdsson and Wachtveitl (52). Finally, at approximately 3–4.5 equivalents, a third neomycin B molecule binds primarily to the upper stem (Figure 4C), thereby increasing the occupancies of all three motifs A, B and C to ∼100% (Figure 2A). Taken together, the NMR and MS data suggest that neomycin B binding to RNA **2** involves three non-identical and—at least for motifs A and B—interdependent sites, which could explain the relatively small differences between *K*_D_ values for motifs A and B (0.4 ± 0.6 and 3.1 ± 1.5 μM) obtained from fitting isothermal titration calorimetry (ITC) (53) data with a two-site model in our previous study (36).

The central role of the hairpin loop and the bulge region of the neomycin sensing riboswitch (binding motif A of RNA **2**, Figures 1D and 2A) for regulatory function has previously been demonstrated in gene reporter assays (38,39). For example, mutating the bulge residues CUU (positions 10–12 of RNA **2** in Figure 2A) towards the sequence of the A-site in the 16 S rRNA of the 30 S ribosomal subunit results in a complete loss of riboswitch function (39) even though neomycin B binds to the A-site motif with high affinity (*K*_D_ = 4 ± 1 nM) (23). Likewise, mutations in the hairpin loop region and the upper stem (C16–G23 in Figure 2) can render the riboswitch inactive (38,54). Mutations in the lower stem are less well studied, but simply replacing the base pairs A6–U30 and U7–A29 with U6–A30 and A7–U29 already reduces the regulatory factor from 7.5 to 5.5 (38). These results support the hypothesis that RNA **2** has more than one binding motif and that an intricate network of inter- and intramolecular interactions enables riboswitch function. To further test this hypothesis, we studied RNA **5** (Table 1) with a single mutation in the upper stem (C13 instead of G13 in RNA **2**) for which no riboswitch activity was detected (38,39). CAD of the 1:1 complexes of RNA **5** with neomycin B showed that the occupancies of binding motifs A and B were, within error limits, the same as for RNA **2**; motif C was not occupied in any of the 1:1 complexes studied here (Supplementary Figure S6). However, the C13 mutation in binding region IV of motif C (RNA **5**) significantly reduced neomycin B binding to motif C in the 1:3 complexes (from ∼100% to ∼60%), which may suggest a possible role of neomycin B binding to motif C in riboswitch function.

With both the MS and the NMR data indicating binding of up to three neomycin B molecules to the riboswitch aptamer RNA **2**, two general questions arise. First, can even more neomycin B molecules bind to RNA **2**, and second, how many neomycin B molecules must be bound for the riboswitch to function? Regarding the first question, we have recorded native ESI MS spectra of RNA **2** with up to five equivalents of neomycin B (Supplementary Figure S7). At the highest neomycin B concentration used, we observed 1:4 complexes, but their dissociation by CAD resulted in loss of one neomycin B molecule, which we interpret as weak and unspecific binding of the fourth neomycin B molecule. To further test the binding specificity of up to three neomycin B molecules to RNA **2**, we performed competition experiments with spermidine (p*K*_a_ 10.7 ± 0.2) (55) in 20-fold excess of neomycin B (Supplementary Figure S8). Because adding 100 μM spermidine increased the solution pH to ∼9.5, and adding CO_2_ to bring it back to ∼7.5 was impractical (spermidine reacts with CO_2_ to form carbamates) (56), we performed control experiments with 100 μM of the ESI additive (57) piperidine (p*K*_a_ 11.1 ± 0.3) (58) at the same pH. Spermidine produced ions with somewhat higher net charges than piperidine, but the fractions of free RNA **2** and its complexes with neomycin B were very similar (Supplementary Figure S8C). Consistent with data for RNA **1** at different pH (36), a decrease in neomycin B protonation with increasing pH reduced aminoglycoside binding to RNA **2** (Supplementary Figures S7D and S8), but higher stoichiometry complexes with neomycin B were still detected even at pH ∼9.5. Importantly, the data in Supplementary Figure S8 show that spermidine does not compete with neomycin B as the fractions of the complexes were similar to those obtained with the ESI additive piperidine. Furthermore, and consistent with the three distinct binding motifs for RNA **2** identified by CAD MS (Figure 1), no indication for specific binding of a fourth neomycin B molecule was found in the NMR spectra. Thus only up to three molecules of neomycin B bind to RNA **2** with demonstrated specificity. Regarding the second question, the NMR data suggest that binding of a single neomycin B molecule already induces the formation of large parts of the functional structure of RNA **2**, and that binding of a second neomycin B molecule merely stabilizes this structure. According to the NMR data, the third neomycin B molecule further reinforces the aptamer structure of RNA **2**. We hypothesize that the riboswitch uses binding motifs A–C to gradually stabilize its aptamer structure in response to increasing concentrations of neomycin B. This could provide the basis for the dose-dependent cellular response mechanism that impacts the scanning machinery for ribosomal translation initiation.

## Conclusions

We demonstrate how native top-down MS in combination with RNA structure prediction can be used to identify multiple binding motifs of a riboswitch aptamer and to relatively quantify their occupancy with the aminoglycoside ligand neomycin B for any given complex stoichiometry. We further show by NMR spectroscopy that the binding of up to three neomycin B molecules is not an artifact from electrospray ionization but can also be observed in solution. Binding of the first neomycin B molecule can take place at two different motifs with similar probability, but in each case stabilizes the entire aptamer structure. Binding of the second and third neomycin B molecules is, however, still specific and further stabilizes and structures the aptamer that controls translation initiation. A single mutation in the upper stem of the riboswitch aptamer that abolishes riboswitch function reduces the occupancy of the binding motif for the third ligand from ∼100% to ∼60% in the 1:3 complexes, supporting the hypothesis that all three neomycin B molecules may play a role in riboswitch function. Overall, the data presented here show that a simple 1:1 binding model with a single binding motif cannot adequately describe neomycin B binding to the riboswitch aptamer, and we speculate that the binding of naturally occurring small molecules to RNA may in general have evolved to a level of complexity that cannot be captured by a single technique. The combination of native top-down MS, RNA structure prediction, and ^1^H NMR spectroscopy in the imino proton region is a powerful approach for future studies of other RNAs and ligand molecules to test the validity of this hypothesis.

## Supplementary Material

gkae224_Supplemental_File

## Data Availability

The data underlying this article are available in the article and in its online supplementary material.
