# Supplementary material for: Resolving the intricate binding of neomycin B to multiple binding motifs of a neomycin-sensing riboswitch aptamer by native top-down mass spectrometry and NMR spectroscopy
Source: Nucleic Acids Res. 2024 Apr 3;52(8):4691–701. doi: 10.1093/nar/gkae224 (PMC11077050; doi:10.1093/nar/gkae224)
Supplement: gkae224_Supplemental_File [file gkae224_supplemental_file.pdf]

Supplementary Data

Resolving the intricate binding of neomycin B to multiple binding motifs of a neomycin-sensing riboswitch aptamer by native top-down mass spectrometry and NMR spectroscopy

Sarah Viola Heel, Fabian Juen, Karolina Bartosik, Ronald Micura, Christoph Kreutz, and Kathrin Breuker*

Institute of Organic Chemistry and Center for Molecular Biosciences Innsbruck (CMBI)
University of Innsbruck
Innrain 80/82, 6020 Innsbruck (Austria)

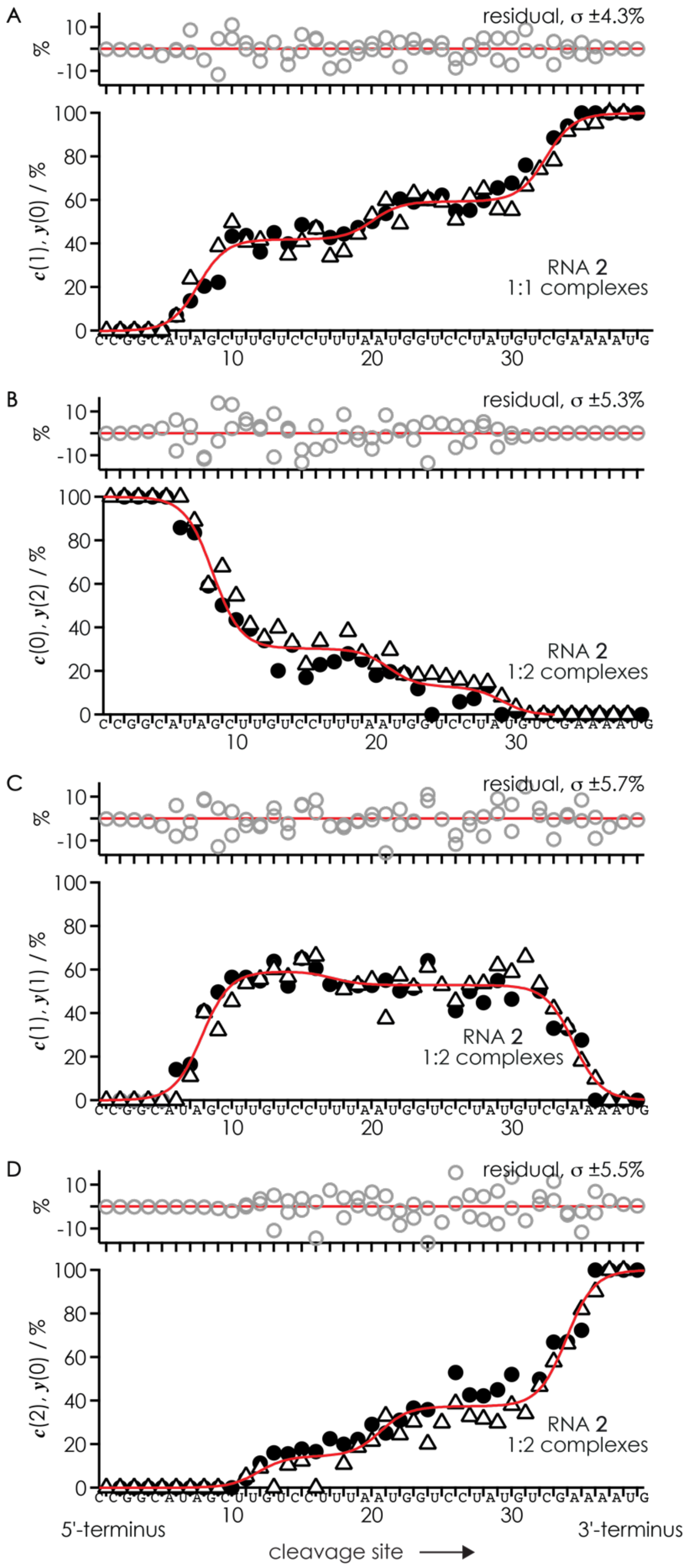

Figure S1: Percentage of c (circles) and complementary y (triangles) fragments with 0, 1, or 2 neomycin B molecules attached versus RNA cleavage site from CAD of A) (RNA **2** + 1·neomycin B - 13H)$^{13-}$ ions and B-D) (RNA **2** + 2·neomycin B - 13H)$^{13-}$ ions; multiple sigmoidal fit functions shown as red lines and residuals with their standard deviations are plotted above each graph. The data were previously published in reference 36, but without the analysis shown here.

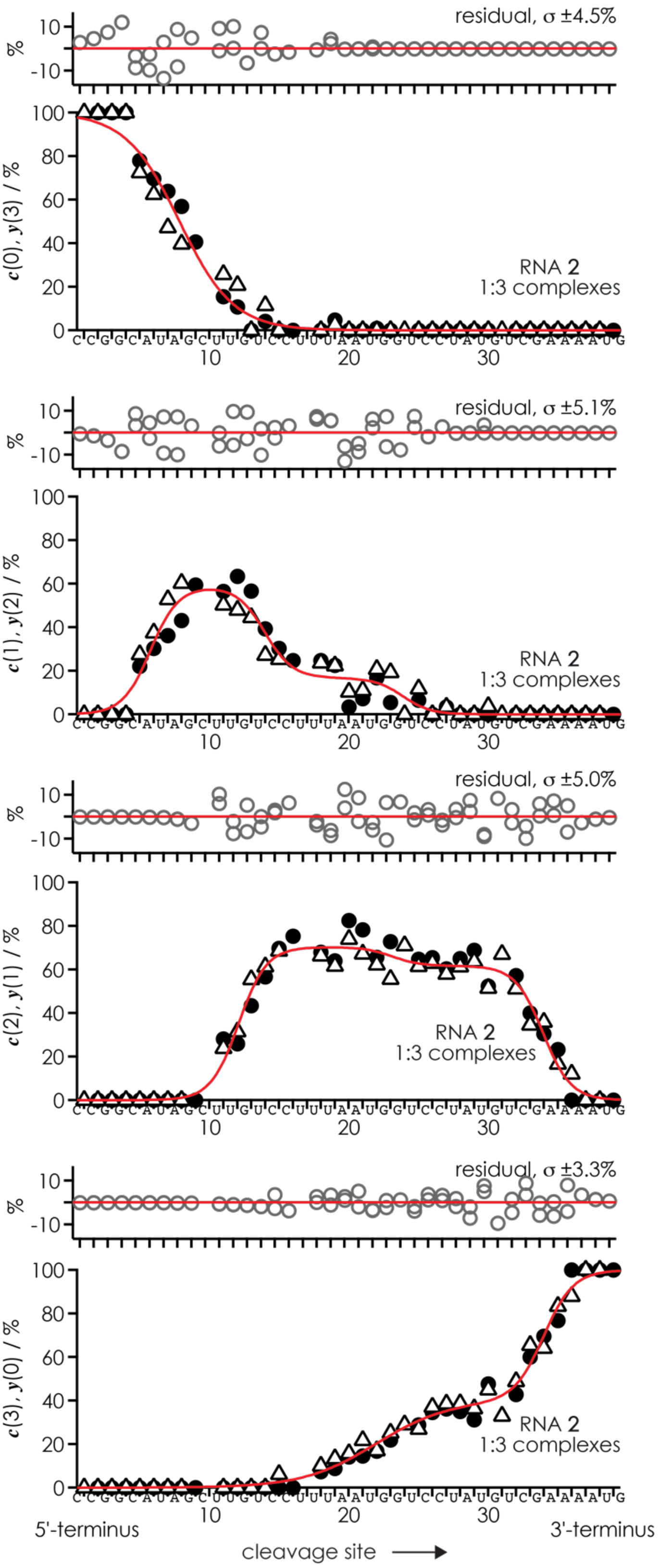

Figure S2: Percentage of c (circles) and complementary y (triangles) fragments with 0, 1, or 2 neomycin B molecules attached versus RNA cleavage site from CAD of (RNA **2** + 3·neomycin B - 13H)$^{13-}$ ions; multiple sigmoidal fit functions shown as red lines and residuals with their standard deviations are plotted above each graph.

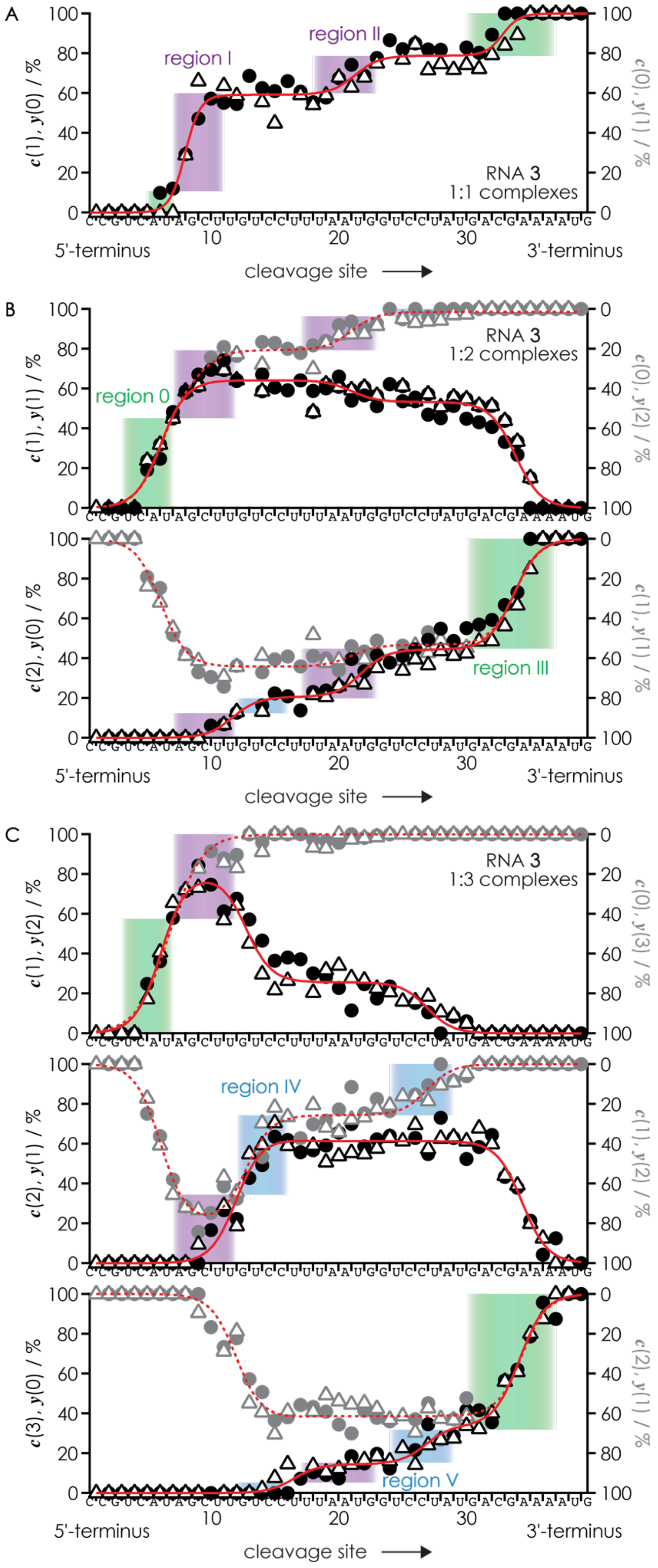

Figure S3. Percentage of ***c*** (circles) and ***y*** (triangles) fragments with 0, 1, 2, or 3 neomycin B molecules attached versus RNA cleavage site from CAD of A) (RNA **3** + 1·neomycin B - 13H)13- ions, B) (RNA **3** + 2·neomycin B - 13H)13- ions, and C) (RNA **3** + 3·neomycin B - 12H)12- ions with multiple sigmoidal fit functions shown as red lines. The data in A) were previously published in reference 36.

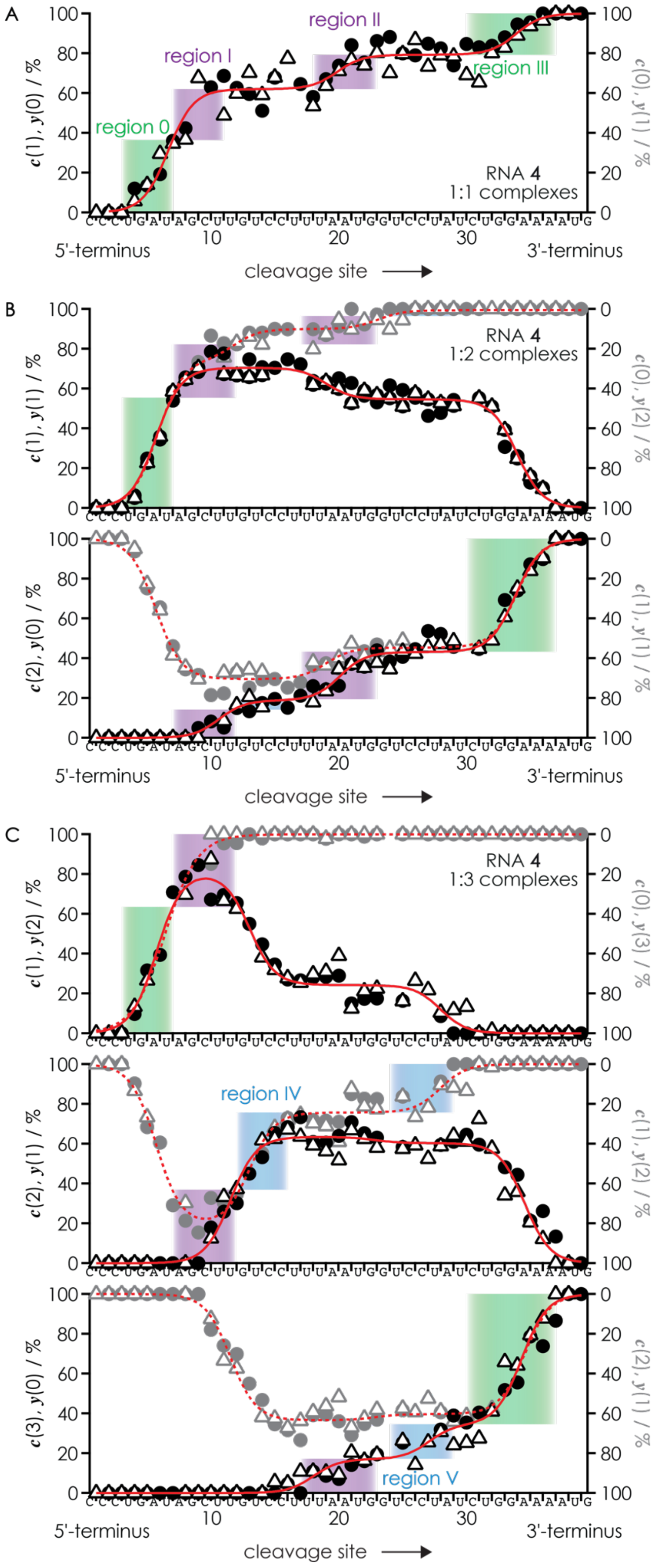

Figure S4. Percentage of ***c*** (circles) and ***y*** (triangles) fragments with 0, 1, 2, or 3 neomycin B molecules attached versus RNA cleavage site from CAD of A) (RNA **4** + 1·neomycin B - 13H)$^{13-}$ ions, B) (RNA **4** + 2·neomycin B - 13H)$^{13-}$ ions, and C) (RNA **4** + 3·neomycin B - 12H)$^{12-}$ ions with multiple sigmoidal fit functions shown as red lines. The data in A) were previously published in reference 36.

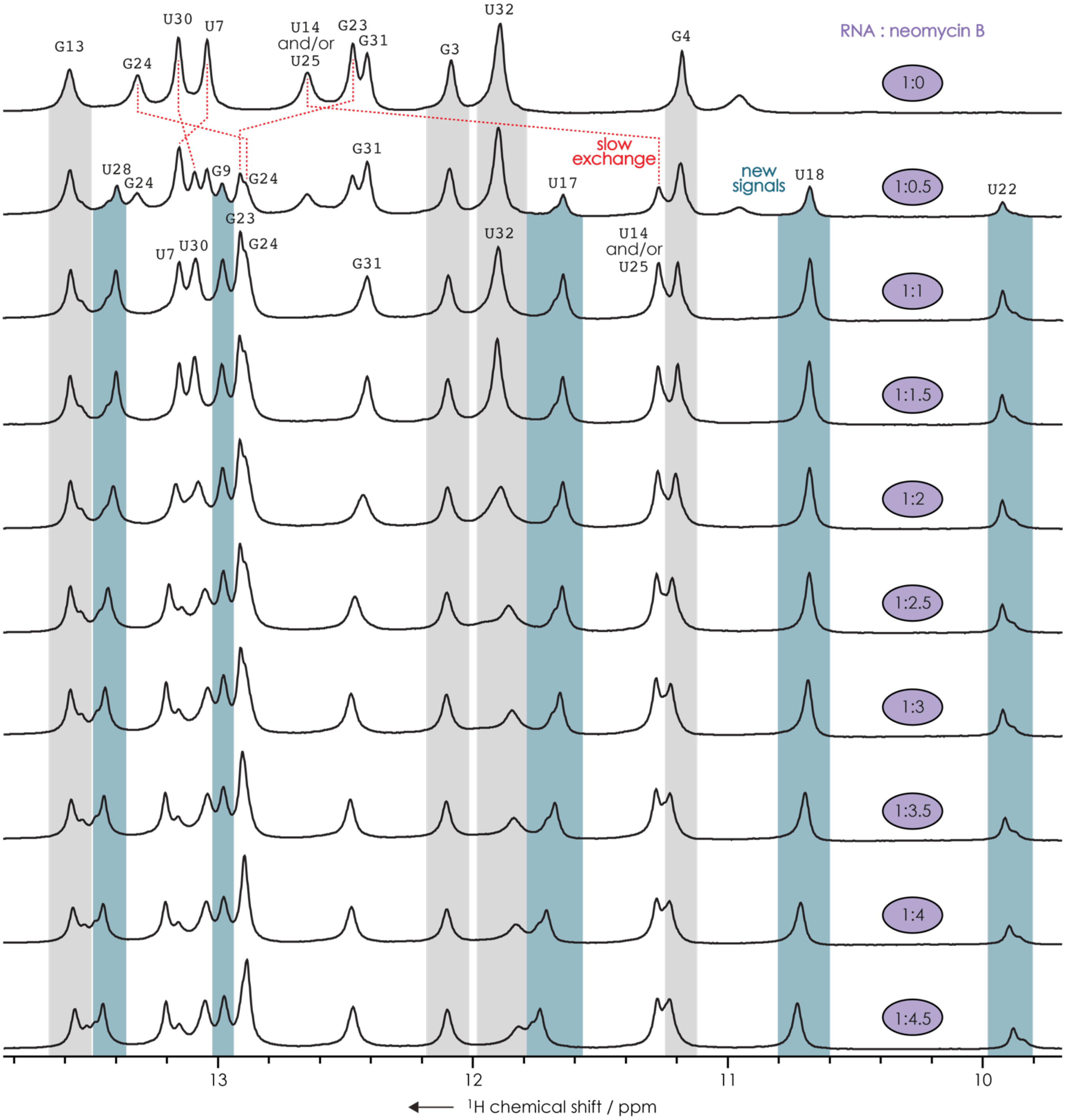

Figure S5. ^{1}H NMR spectra in the imino proton region for RNA **2** (280 μM) titrated with up to 4.5 equivalents of neomycin B (62.5 mM) in NMR buffer (15 mM sodium phosphate, 25 mM sodium chloride, 10% vol/vol D_2O with the pH adjusted to 6.5 by addition of sodium hydroxide) at 15 °C. Signals were assigned as described in the experimental section.

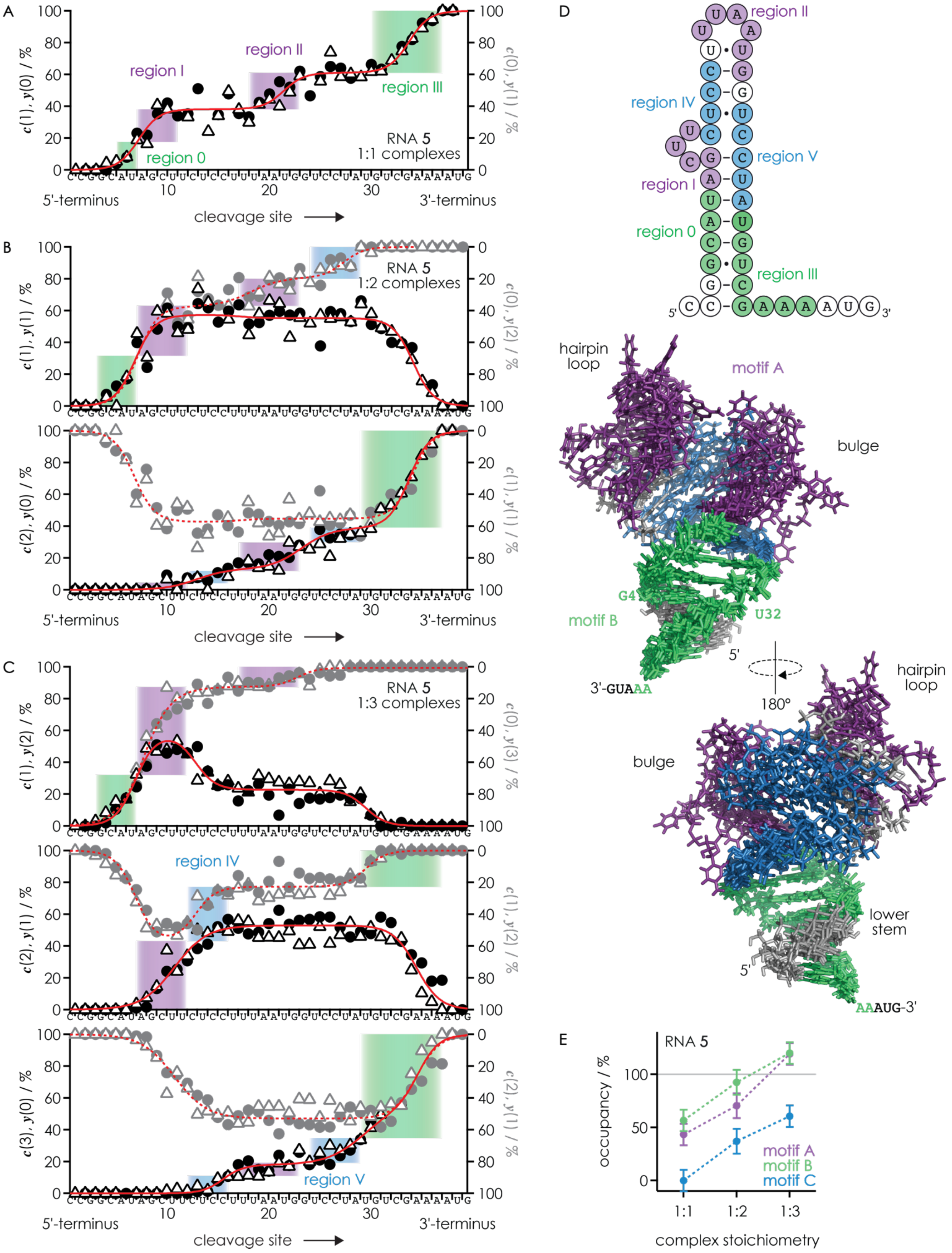

Figure S6. Percentage of c (circles) and y (triangles) fragments with 0, 1, 2, or 3 neomycin B molecules attached versus RNA cleavage site from CAD of A) (RNA **5** + 1·neomycin B - 13H)$^{13-}$ ions, B) (RNA **5** + 2·neomycin B - 13H)$^{13-}$ ions, and C) (RNA **5** + 3·neomycin B - 12H)$^{12-}$ ions; D) binding regions of the 1:2 and 1:3 complexes mapped onto 5 structures predicted for free RNA **5** (excluding `A36-G40`) by the MC-fold|MC-Sym pipeline; E) occupancy of motifs A-C for the 1:1, 1:2, and 1:3 complexes of RNA **5**.

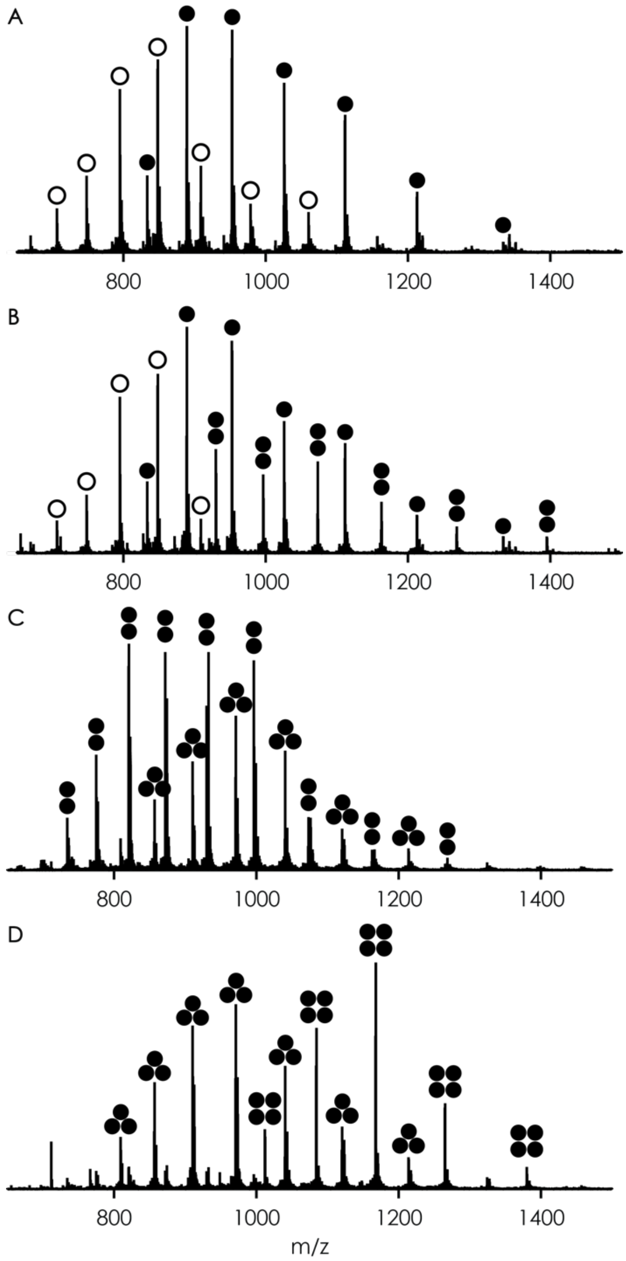

Figure S7: Native ESI of RNA **2** (1 μM) and A) 1 μM, B) 2 μM, C) 3 μM, and D) 5 μM neomycin B in 9:1 H_2O/CH_3OH with 50 mM ammonium bicarbonate and 0.25 mM piperazine (pH ~7.5) incubated for 3 hours shows signals of free RNA (O), and 1:1 (●), 1:2 (●●),1:3 (●●●), and 1:4 (●●●●) RNA/neomycin B complexes.

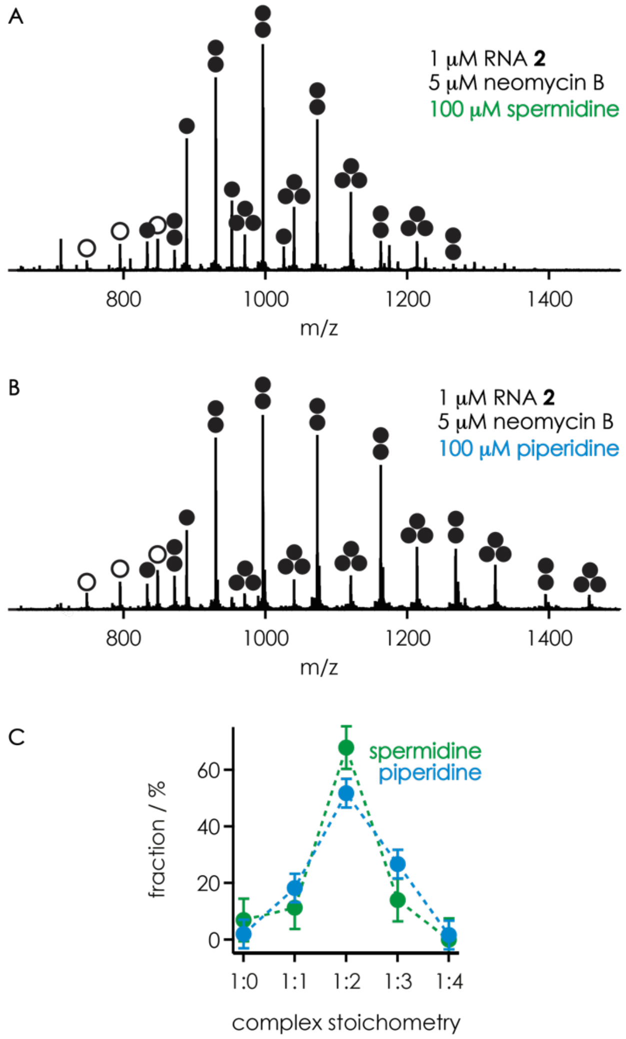

Figure S8: Native ESI of RNA **2** (1 µM) and 5 µM neomycin B in 19:1 H_2O/CH_3OH with 100 mM ammonium bicarbonate and 100 µM A) spermidine and B) piperidine (both at pH ~9.5) incubated for 1 hour shows signals of free RNA (O), and 1:1 (●), 1:2 (●●), 1:3 (●●●), and 1:4 (●●●●) RNA/neomycin B complexes. The net charges of the ions in the spectrum with spermidine (A) are somewhat higher than in the spectrum with piperidine (B), but the fractions of free RNA, 1:1, 1:2, and 1:3, and 1:4 RNA/neomycin B complexes were not significantly different for spermidine and piperidine (C). Fractions indicated in C are average values with error bars (two standard deviations) from triplicate experiments.
